# The effect of vitamin D deficiency on urinary incontinence during third trimester pregnancy

**DOI:** 10.1097/MD.0000000000036044

**Published:** 2023-11-10

**Authors:** Sezer Gul, Huseyin Aydogmus, Caglasu Keles, Serpil Aydogmus, Mustafa Sengul

**Affiliations:** a Izmir Katip Celebi University Atatürk Training and Research Hospital, Department of Obstetrics and Gynecology Izmir, Karabağlar/İzmir, Turkey; b Izmir Katip Celebi University Faculty of Medicine, Department of Obstetrics and Gynecology Izmir, Karabağlar/İzmir, Turkey.

**Keywords:** pregnancy, urinary incontinence, vitamin D

## Abstract

Urinary incontinence (UI) is a common problem which is associated with impaired quality of life. Vitamin D plays a crucial role for pelvic floor muscle function. The aim of this study was to investigate the effect of vitamin D deficiency on UI in pregnant women in the third trimester of pregnancy. All pregnant women at > 28 weeks of gestation who were followed in the gynecology and obstetrics outpatient clinic were screened. The patients were assessed for UI during routine follow-up. The Incontinence Severity Index was used to determine the severity of UI. A total of 210 patients were included as the study group and 40 patients were included as the control group. Both groups were compared based on the International Incontinence Severity Index scores. Of the patients, 40% had a history of UI and 84% had vitamin D deficiency. Pregnant women with vitamin D deficiency had statistically significant UI, compared to pregnant women in the control group. The severity of UI was also significantly higher in the patients with vitamin D deficiency. Urinary incontinence is significantly associated with vitamin D deficiency in pregnant women.

## 1. Introduction

Urinary incontinence (UI) is a serious health issue associated with anxiety, depression, and difficulties at work that contribute to social isolation.^[1]^ Pregnancy and vaginal delivery are major risk factors for UI.^[2]^ The mean prevalence of UI during pregnancy is 41%.^[3]^ Risk factors for the development of UI during pregnancy include weight gain increased uterine volume, increase intra-abdominal pressure, pressure on the pelvic floor and bladder, and enlargement of the genital hiatus with increasing parity.^[4,5]^ The peripartum period is frequently the first time women experience UI.^[2]^ A study has shown that UI during pregnancy significantly increases the risk of developing UI within 12 years of delivery.^[6]^

Vitamin D influences both male and female reproductive tissues via receptors. Via receptors are found in the hypothalamus, ovaries, testes, endometrium, and placenta. Therefore, vitamin D plays an important role in human reproductive physiology.^[7]^

Vitamin D is important for couples fertility, and Vitamin D deficiency could be a cause of infertility and affect the success of assisted reproductive techniques.^[8,9]^

Vitamin D affects decidualization, implantation, and hormonal and immune responses in the placenta.^[10]^ Vitamin D deficiency and insufficiency may have a significant negative effect on pregnancy outcomes. Several gestational complications, such as gestational diabetes, gestational hypertension, premature rupture of membranes, and premature delivery may occur due to vitamin D deficiency.^[11]^

Vitamin D also plays a crucial role in pelvic floor muscle functions. Several studies have demonstrated that vitamin D deficiency is associated with UI in nonpregnant women.^[12,13]^ In addition, vitamin D deficiency during pregnancy has been linked to postpartum pelvic floor muscle dysfunction.^[14]^ To the best of our knowledge, only 1 study investigating the association between vitamin D deficiency and UI during pregnancy. Stafne et al^[15]^, in their study including 851 healthy pregnant women between 18 and 22 weeks of pregnancy, reported that UI in pregnant women with vitamin D deficiency was significantly more common with a higher prevalence.

In this study, we aimed to investigate the prevalence of UI and examine the relationship between vitamin D deficiency and UI in full-term pregnant women.

## 2. Methods

### 2.1. Study design and study population

**Figure 1.**
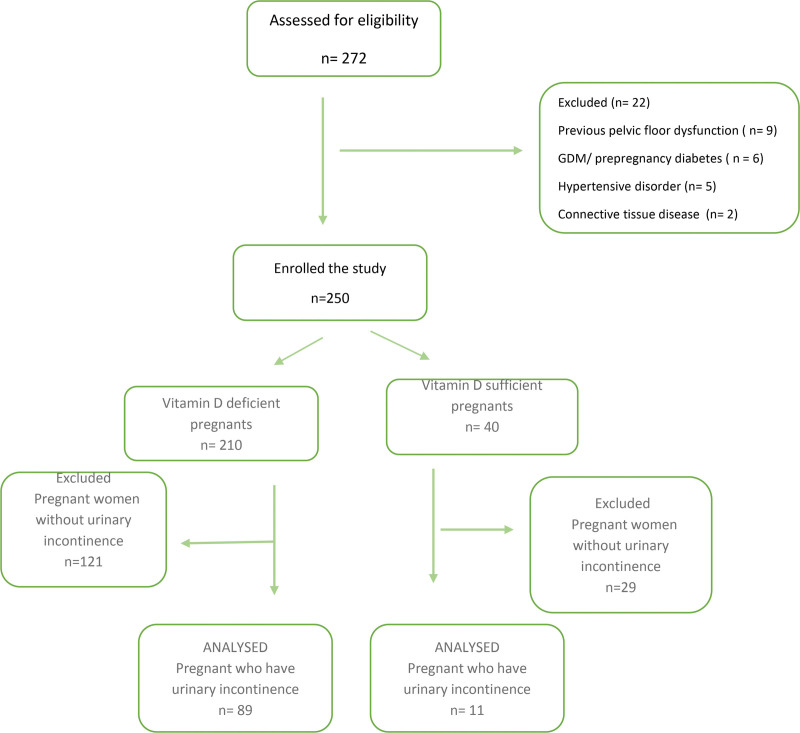
Study flow chart.

This single-center, cross-sectional study was conducted at the Department of Gynecology and Obstetrics of a tertiary care center between January 2021 and November 2021. The study included 250 healthy pregnant women over the age of 18 years with a singleton pregnancy over 28 weeks who were admitted to our clinic (Fig. 1). Those with gestational or pregestational diabetes mellitus, hypertension or connective tissue disease, a history of pelvic floor surgery, cognitive disorders or neurological diseases, acute urinary tract infection, and lost to follow-up were excluded from the study. All patients were informed of the nature of the study, and a written informed consent was obtained from all patients. The study was approved by the Institutional Ethics Committee (IRB:89/2020) and conducted in accordance with the principles of the Declaration of Helsinki.

### 2.2. Data collection and data analysis

Demographic data, obstetric histories, smoking, alcohol use, drug use, and chronic medical conditions were recorded. During antenatal follow-up, pregnant women who were at ≥ 28 weeks of pregnancy were questioned about their UI history. Subjective incontinence was deemed positive if participants experienced UI at least once during pregnancy and negative if they did not. The International Incontinence Severity Index (ISI), developed by Sandvik et al^[16]^, whose validity and reliability studies in the Turkish population were conducted by Hazar et al^[17]^, was used to determine the severity, frequency, and type of UI in pregnant women with UI throughout the study. In the questionnaire, each question was assigned a point value between 1 and 4. These scores were multiplied to yield an incontinence severity score (minimum, 1; maximum, 12) (Appendix 1: ISI index). The ISI includes 2 subitems: “How frequently do you urinate?” and “How much urine do you leak per day?”. The ISI score was calculated by multiplying the number of questions for each item. The score was determined by grouping 1 and 2 as mild, 3 and 6 as moderate, 8 and 9 as severe, and 12 as extremely severe (Appendix 1).

During the initial evaluation, 5 cc of venous blood was drawn from the pregnant women, centrifuged, and stored at −80°C until analysis. By requesting a thorough urinalysis and, if necessary, urine culture, an acute urinary infection was ruled out. Serum vitamin D levels were measured after the completion of 250 cases. Serum 25 (OH) D3 concentrations were measured using enzyme-linked immunosorbent assay (Global Diagnostics and Medical Solutions KAP1971/GDMS, 13E27/2, Mortsel, Belgium). Serum 25 (OH) D3 concentrations < 20 ng/mL were classified as deficient (intra-assay coefficient variability,5.7%; inter-assay coefficient variability, 4.7%). The study group consisted of 210 women with insufficient vitamin D levels, whereas the control group consisted of 40 women with adequate vitamin D levels. Both groups were compared based on ISI scores.

### 2.3. Statistical analysis

Statistical analysis was performed using the SPSS version 26.0 software (IBM Corp., Armonk, NY). Descriptive data are presented as mean ± standard deviation or median (Q1–Q3) for continuous variables and as numbers and frequencies for categorical variables. The normality of the data distribution for numerical variables was assessed using the Shapiro–Wilk test and Q-Q charts. Levene test was used to assess homogeneity of variance. For normally distributed variables, a 2-sample independent *t* test was used to compare the groups, whereas the Mann–Whitney U test was used for non-normally distributed variables. Fisher chi-squared test was used to compare categorical variables. If the Fisher exact test result was significant, the 2-ratio Bonferroni-corrected *z* test was performed to analyze the differences between the groups. Statistical significance was set at *P* value < .05.

## 3. Results

Baseline demographic characteristics of the participants are presented in Table 1. The mean age of the study group and control group was 27.76 ± 5.9 years and 27.35 ± 4.60 years, respectively. The mean body mass index was 29.37 ± 5.15 kg/m^2^ and 27.82 ± 3.93 kg/m^2^, respectively. The mean gestational week was 33.86 ± 4.11 weeks, respectively. Both groups were similar in terms of age, body mass index, and gestational age.

**Table 1:** Demographic features of participants.

	Vitamin D deficient pregnants (n:210)	Vitamin D sufficient pregnants (n:40)	*P* value
Mean age (yr)	27.76 ± 5.9	27.35 ± 4.60	.67
Mean body mass index (kg/m2)	29.37 ± 5.15	27.82 ± 3.93	.07
Mean gestational age (wk)	33.86 ± 4.11	33 ± 3.42	.21
Mean gravity	2.58 ± 1.42	2.07 ± 1.24	.02
Mean parity	1.28 ± 1.17	0.82 ± 0.78	.03
Mean vitamin D level	8.99 ± 3.85	29.19 ± 9.68	.02
Delivery method			.04
Nullipara	60 (28.6%)	15 (37.5%)	
Vaginal birth	96 (45.7%)	9 (22.5%)	
Cesarean section	54 (25.7%)	16 (40%)	

A total of 40% (100) of all the pregnant women who participated in the study had UI at any time during pregnancy. On comparing the 2 groups based on subjective incontinence reporting, 42.4% (n = 89) of the pregnant women in the study group and 27.5% (n = 11) of the pregnant women in the control group experienced UI at least once during pregnancy (Table 2).

**Table 2:** Incontinence frequency and severity of nulliparous women.

	Vitamin D deficient nulliparas (n:52)	Vitamin D sufficient pregnants (n:15)	*P* value
Nulliparas with urinary incontinence	21 (40.4%)	1 (6.7%)	0.02*
Mean incontinence sevirity index (ISI) score	4.11 ± 2.02	2.4 ± 1.72	0.03*

* Pearson Chi-Square test.

A total of 42.4% of pregnant women with vitamin D deficiency had UI compared to 27,5% of pregnant women in the control group (*P* = .026). The ISI scores of the pregnant women in the study group were also significantly higher than those of the control group (Table 2).

## 4. Discussion

In the present study, we investigated the effect of vitamin D deficiency on UI in women in the third trimester of pregnancy. Our study results showed that 84% of the women had vitamin D deficiency, while 40% had a history of urinary incontinence. Pregnant women with low vitamin D levels had significantly more UI issues than those with normal vitamin D levels.

Vitamin D deficiency during pregnancy is a global health issue.^[18]^ In a recent study conducted in Turkey, the prevalence of vitamin D deficiency in individuals aged 2152 years was estimated at 40% to 96.6%.^[19]^

The pelvic floor muscles and bladder contain vitamin D receptors. Therefore, vitamin D deficiency may affect pelvic floor function and urinary continence.^[20,21]^ It has been shown in the literature that the prevalence of urinary incontinence is lower in patients with high vitamin D levels.^[12,13,22,23]^ During the postpartum period, a significant decrease in pelvic floor muscle strength has been observed in patients with vitamin D deficiency. In addition, the incidence of UI was higher among women with vitamin D deficiency, although the difference was not statistical significance.^[14]^ A recent study showed that vitamin D replacement reduced the severity of SUI in women with vitamin D deficiency.^[24]^

To the best of our knowledge, only 1 study has investigated the relationship between vitamin D deficiency and UI during pregnancy in the literature.^[19]^ In this study, Stafne e al. found that 27% of healthy, nulliparous, mid-trimester pregnant women living in Northern Europe experienced UI, particularly stress incontinence, and the prevalence of UI was higher in pregnant women with vitamin D deficiency.

In the present study, consistent with the literature, but slightly higher than that reported by Stafne et al, we determined that the prevalence of UI in pregnant women living in our region was 40%. To evaluate the relationship between vitamin D deficiency and UI during pregnancy, we assessed the presence and severity of UI in pregnant women with vitamin D deficiency. We aimed to determine the serum vitamin D levels of women in the third trimester of pregnancy to reduce the effect of factors such as nutrition, dressing style, and supplementation status on vitamin D levels in pregnant women. We found that both the presence and severity of UI were significantly higher in pregnant women with vitamin D deficiency than in the control group. Considering the high prevalence of pelvic floor disorders and incontinence in the female population with vitamin D deficiency, we believe that it is reasonable to have similar results in pregnant women with vitamin D deficiency.

Nonetheless, this study had some limitations. The main limitations of this study its cross-sectional design and relatively small sample size. On the other hand, the main strength of our study is that it investigated both the current UI status and UI experience during pregnancy in pregnant women, and it is one of the first studies to examine the association between vitamin D deficiency and UI during pregnancy.

## 5. Conclusion

In conclusion, vitamin D deficiency is significantly associated with the development of UI in pregnant women. Although these results are consistent with those in the existing literature, further large-scale prospective studies are required to investigate the association between vitamin D deficiency and UI in pregnant women.

## Author contributions

**Conceptualization:** Sezer Gul.

**Data curation:** Sezer Gul, Huseyin Aydogmus, Caglasu Su, Serpil Aydogmus.

**Formal analysis:** Sezer Gul, Huseyin Aydogmus.

**Investigation:** Sezer Gul, Huseyin Aydogmus.

**Methodology:** Sezer Gul, Huseyin Aydogmus.

**Resources:** Sezer Gul.

**Supervision:** Mustafa Sengul.

**Validation:** Sezer Gul.

**Writing – original draft:** Sezer Gul, Huseyin Aydogmus, Mustafa Sengul.

**Writing – review & editing:** Huseyin Aydogmus, Serpil Aydogmus, Mustafa Sengul.
